# Parent and friend support and communication as correlates of sexual and gender minority alcohol use in sexual contexts

**DOI:** 10.3389/fpsyg.2026.1932437

**Published:** 2026-09-30

**Authors:** Sarah E. Harper, Tina Zawacki

**Affiliations:** Psychology Department, University of Texas at San Antonio, San Antonio, TX, United States

**Keywords:** alcohol use, emerging adult, LGBTQ+, sex communication, sexual and gender minorities, sexual health risk, social support

## Abstract

**Purpose:**

Emerging adults who identify as sexual and gender minorities (SGM) often report greater alcohol use and adverse sexual health experiences compared to cisgender heterosexual (CH) peers. This study investigated whether participants’ identity as either SGM or CH was related to drinking in sexual contexts (DISC) through its relationship with levels of social support, supportive communication, and sex communication from parents and friends.

**Methods:**

Cross-sectional survey responses were collected on-line during November 2024–May 2025 from a convenience sample of 674 university students in the USA.

**Results:**

Two path models were conducted using the PROCESS macro in SPSS, one examining parent variables, and the other examining friend variables. Each model specified one predictor (identity: CH or SGM), one outcome (DISC), and four parallel mediators (social support [parent/friend]; general communication [parent/friend], open sex communication [parent/friend]; lack of sex communication [parent/friend]. The indirect path of identity to DISC was significant via parent lack of sex communication (*b* = 0.11, SE = 0.06, 95% CI [0.01, 0.24]) and friend open sex communication (*b* = 0.11, SE = 0.04, 95% CI [0.04, 0.22]), but not via parent/friend social support and general communication, parent open sex communication, and friend lack of sex communication. Compared to CH participants, SGM participants reported significantly more open friend sex communication and parent lack of sex communication which were associated with significantly higher levels of DISC.

**Conclusion:**

These findings highlight the importance of DISC within the conversation of SGM sexual health and the need for family and peer-based interventions.

## 1 Introduction

Sexual and gender minority (SGM) individuals experience elevated risk of sexually transmitted infections (STIs), with higher rates reported among gay men, bisexual men, bisexual women, and transgender women compared to cisgender heterosexual (CH) individuals (Centers for Disease Control and Prevention, 2024a; Centers for Disease Control and Prevention, 2024b). In addition, SGM individuals face elevated rates of sexual victimization (Coulter et al., 2017; Kammer-Kerwick et al., 2021), with higher rates reported by transgender women (Abern et al., 2022; James et al., 2016), nonbinary individuals (James et al., 2016), lesbian women (Canan et al., 2021) and bisexual women (Eisenberg et al., 2021) than by CH individuals. Although sexual health threats vary across SGM subgroups, nearly every subgroup experiences elevated risk of either sexual victimization or STI exposure. This overarching disparity in sexual health highlights the importance of investigating factors among SGM individuals. The present study investigates the roles of social support, general supportive communication, and sex communication in the relationship between SGM identity and the sexual health risk factor of alcohol use during sexual encounters.

Over 50% of sexual violence victims report having consumed alcohol at the time of the assault (Read, 2025), and drinking increases event-level sexual risk-taking such as condomless sex (Berry and Johnson, 2018; Shuper et al., 2017). Research suggests that drinking is a particularly important factor in both of these negative sexual health outcomes among SGM individuals. General alcohol use is elevated among SGM emerging adults compared to CH peers (Centers for Disease Control and Prevention, 2024c; Li et al., 2022) and is correlated with their sexual risk behavior (Li et al., 2022; Shuper et al., 2017), and sexual violence experience (Kiekens et al., 2021; Marcantonio and Willis, 2022). Nonetheless, alcohol-involved sexual behavior remains understudied among SGM individuals. The present study addresses this research gap by focusing on alcohol use during sexual encounters.

Ample research has shown that emotional social support—feeling that one can depend on people in one’s life, typically parents and friends, for acceptance, compassion, empathy, trust, and reassurance – is associated with positive health outcomes (Cutrona, 1990; Yeo et al., 2025). In studies focusing on sexual risk among CH adolescents, emotional support from parents is a consistent protective factor (Dutra et al., 1999; Henrich et al., 2006), and interventions have leveraged parent support to reduce adolescent sexual risk-taking (Guilamo-Ramos et al., 2023; Widman et al., 2019). Further, the association between parent support in adolescence and health outcomes persists into emerging adulthood (Ford et al., 2023; Saint-Eloi et al., 2024; Steiner et al., 2019).

However, SGM adolescents experience unique challenges regarding parent emotional support. Minority Stress Theory (MST) posits that stressors related to SGM identity, including lack of emotional support, contribute to adverse health outcomes including alcohol use and sexual victimization (Ison et al., 2025; Hinds et al., 2024). Family rejection in particular has been linked to increases in minority stress outcomes such as mental health challenges and sexual risk-taking (Xu et al., 2026). Lack of family support about SGM identity can lead to family tension (Robinson, 2018), and degrees of discomfort and avoidance can create barriers for SGM adolescents to emotionally connect with their parents (Ghosh, 2020). Following “coming out” to family, some SGM adolescents experience increases in health risk-taking (Ryan et al., 2010) including sexual risk-taking (Feinstein et al., 2017; Glick and Golden, 2014). In accordance, some literature suggests that parent support may act as a less effective protective factor for sexual risk-taking for SGM adolescents than for CH peers (Ryan et al., 2010; Thoma and Huebner, 2014). The present study further examines the role of general parent support and communication in sexual risk-related behavior of SGM and CH emerging adults and investigates specific forms of communication that may play a role such as sex communication.

Ghosh (2020) found among lesbian and gay adolescents that even when parents are accepting of a child’s sexual minority identity, they may avoid discussing sex with their child, potentially weakening the protection from health risk that familial support typically exerts for emerging adults. Qualitative research on parent sex communication with SGM individuals has similarly identified themes of discomfort and lack of knowledge as barriers to effective communication and subsequent reductions in sexual risk-taking by SGM emerging adults (Bond et al., 2023; Newcomb et al., 2019; McKay and Fontenot, 2020). Mixed results exist from quantitative data on parental sex communication mostly focused on sexual minority men; some studies indicate that high-quality sex communication shows a positive association with risk reduction behaviors (Bouris et al., 2015; McKay and Fontenot, 2020) while other studies do not (Thoma and Huebner, 2014).

Most published sexual risk-taking interventions have not evaluated the effectiveness of improving parental sex communication among SGM emerging adults (Widman et al., 2019; McKay and Fontenot, 2020). Experts have called for increased research on the influence of families on SGM health in order to inform intervention development (Newcomb et al., 2019). The present study expands current research in this area by further investigating the relationship between specific aspects of parent sex communication and sexual risk-related behavior among SGM emerging adults.

Within CH samples, friend emotional support has shown a limited beneficial relationship with sexual risk-taking as compared to parent support (Bruederle et al., 2019; Ndlovu and Moyo, 2026). Henrich et al. (2006) found in a predominantly CH sample of adolescents that friend support only acted as an amplifier of significant parent support associations. Supportive parental relationships, however, are not always present in SGM adolescents’ lives. Research also suggests that friends contribute more than parents to sexual minority adolescents’ emotional support related to their sexual identity (Doty et al., 2010; Jamil et al., 2009). Sexual and gender minority emerging adults may seek support from friends as an alternative to scarce parental support or to mitigate the minority stress caused by insufficient parental support. The present study builds on this research by delineating friends and parents as providers of support and assessing their separate relationships with sexual risk-related behavior.

Whereas the relationships of friend and parent social support and communication with lower sexual risk experience have been widely investigated among heterosexual and cisgender emerging adults, their relationships among SGM individuals remain less clear. In addition, sexual activity during alcohol use shows evidence of being an important sexual risk factor for SGM individuals, yet relatively few studies have examined it. This study addresses these research gaps by investigating whether SGM and CH emerging adults report receiving different patterns of general emotional support, general supportive communication, and sex-specific communication from parents and friends, and in turn, how these patterns relate to participants’ level of alcohol use in sexual situations. This study examines SGM identity as a single group rather than several separate identity groups. This approach is based on the presence of sexual health threats across subgroups, as well as minority stress such as lack of family support shared across subgroups (Li et al., 2022; Scroggs and Rosenberger, 2024). A trade-off of this approach is that we are unable to make subgroup-specific inferences based on our analyses.

We hypothesized that SGM identity would relate to drinking in sexual contexts (DISC) through its relationship with levels of general emotional support, general supportive communication, and sex communication provided by parents and friends. In terms of parent support and utilizing a Minority Stress Theory perspective, we hypothesized that compared to CH participants, SGM participants would report experiencing lower levels of parent general emotional support, parent general supportive communication, and less effective parent sex communication, and in turn, lower parental emotional support, communication, and effective sex communication would be associated with higher levels of DISC.

Based on research suggesting that SGM emerging adults may seek support from friends as an alternative to limited parental support or to mitigate the minority stress caused by lacking parent support, we hypothesized that SGM participants would report experiencing higher levels of friend emotional support, friend supportive communication, and effective sexual friend communication. In turn, higher friend emotional support, communication, and effective sex communication would be associated with lower levels of DISC.

## 2 Materials and methods

### 2.1 Participants

Participants were 674 college students whose mean age was 19.57 years (SD = 2.49, *Mode* = 19.00, *Median* = 19.00, *Range* = 18–48). Most participants (69.6%) were 18 or 19 years old. One quarter (24.6%) of the sample identified with a SGM identity; 75.4% identified as CH. Participants reported the specific SGM sexual identities of bisexual (*n* = 74), pansexual (*n* = 13), gay/lesbian (*n* = 26), queer (*n* = 11), asexual (*n* = 4), questioning (*n* = 13), another/multiple identity but did not specify (*n* = 24). Participants reported the specific SGM gender identities of non-binary (*n* = 8), trans woman (*n* = 2), trans man (*n* = 5), genderqueer/gender expansive (*n* = 13), another identity/multiple identity but did not specify (*n* = 6). Among participants who reported drinking alcohol in the past month (DDQ; Collins et al., 1985), average monthly alcohol consumption was 19.65 drinks (SD = 22.16) and did not differ between CH and SGM participants, *F*(1,472) = 0.036, *p* = 0.850. The number of non-drinking participants also did not differ between SGM and CH participants, *X*^2^ = (1,674) = 0.004, *p* = 0.952. All participants reported at least one instance of dating, sexual intercourse, or sexual contact in their lifetime. Table 1 contains sample demographics.

**Table 1:** Sample characteristics and descriptive results.

Variables	*n* (%)
Gender identity	
Cisgender woman	384 (57.0)
Cisgender man	254 (38.0)
Non-binary	8 (1.2)
Trans woman	2 (0.3)
Trans man	5 (0.7)
Genderqueer/gender expansive	13 (1.9)
Another identity/multiple	6 (0.9)
Sexual identity	
Heterosexual	509 (75.5)
Bisexual	74 (11.0)
Pansexual	13 (1.9)
Gay/lesbian	26 (3.9)
Queer	11 (1.6)
Asexual	4 (0.6)
Questioning	13 (1.9)
Another identity/multiple	24 (3.6)
Ethnicity	
Latinx/Latine American or Hispanic	363 (53.9)
European American or Non-Hispanic White	123 (18.2)
African American or Black	58 (8.6)
Asian or Pacific Islander	48 (7.1)
Arabic or Middle Eastern	9 (1.3)
Native American or American Indian	6 (0.9)
Multiracial	65 (9.6)
Other	2 (0.3)
Religious affiliation	
Catholic	251 (37.2)
Non-denominational Christian	98 (14.5)
Protestant	54 (8.0)
Fundamentalist	52 (7.7)
Islamic	17 (2.5)
Hindu	7 (1.0)
Buddhist	5 (0.7)
Jewish	4 (0.6)
No religious affiliation	145 (21.5)
Other	25 (3.7)
Unspecified	16 (2.4)

Demographics may not sum to 100% due to rounding estimation.

### 2.2 Procedures

An on-line survey study using the Qualtrics platform collected cross-sectional, self-report data during November 2024 to May 2025 from a convenience sample of students attending a large, public university in the southwestern USA. Participants were recruited via the psychology department subject pool and remunerated with class credit. Qualtrics data quality features were utilized to screen out duplicate and bot-generated responses. Participants provided electronic informed consent. All study procedures and materials were approved by the university’s IRB (approval: FY23-24-359) on October 15, 2024.

### 2.3 Measures

Mean scores of multi-item scales were created with higher values representing higher level or frequency of the variable. Participants answered all support/communication measures once regarding parents and once regarding friends. Descriptive statistics including Cronbach’s alpha and correlations for all study variables are presented in Table 2.

**Table 2:** Correlations and descriptive statistics for continuous variables.

Variable	1	2	3	4	5	6	7	8	9
1. DISC	1.00								
2. Parent support	−0.00	1.00							
3. Parent general communication	−0.00	0.77^**^	1.00						
4. Parent lack of sex communication	0.09^*^	−0.12^**^	−0.22^**^	1.00					
5. Parent openness of sex communication	−0.03	0.38^**^	0.49^**^	−0.28^**^	1.00				
6. Friend support	0.02	0.36^**^	0.24^**^	0.00	0.09^*^	1.00			
7. Friend general communication	0.02	0.27^**^	0.23^**^	0.04	0.09^*^	0.78^**^	1.00		
8. Friend lack of sex communication	0.04	0.06	0.04	0.25^**^	−0.02	−0.02	0.01	1.00	
9. Friend openness of sex communication	0.10^**^	0.07	0.04	0.15^**^	0.04	0.36^**^	0.44^**^	−0.00	1.00
Mean	2.76	4.24	3.68	2.83	2.74	4.31	4.11	2.22	4.03
Standard deviation	2.87	0.85	1.01	1.59	1.09	0.77	0.82	1.23	0.89
Observed range	1–13.3	1–5	1–5	1–5	1–5	1–5	1–5	1–5	1–5
Skew	2.01	−1.36	−0.58	0.18	−0.02	−1.46	−0.84	0.78	−1.26
Kurtosis	3.78	1.63	−0.42	−1.53	−0.83	2.41	0.38	−0.40	1.98
Cronbach’s alpha	0.88	0.97	0.93	0.91	0.91	0.98	0.94	0.89	0.92

Correlations are presented with pairwise deletion. ^**^p < 0.001; ^*^p < 0.05. DISC, Drinking in Sexual Contexts.

#### 2.3.1 Demographics

Age was reported by open-ended response (“What is your age?”). Religious affiliation (“What is your religious affiliation?”) and ethnicity (“How would you describe your ethnic background?”), were reported by selecting categories presented in Table 1.

#### 2.3.2 Sexual and gender identity

Participants reported their current gender identity and sexual identity by selecting from the options presented in Table 1. Participants could select more than one response for both items. For data analyses, SGM participants were categorized as individuals selecting a gender identity other than cisgender woman or man or selecting any sexual identity other than heterosexual. Cisgender heterosexual participants were categorized as individuals who selected only the identities of heterosexual and cisgender woman or man.

#### 2.3.3 Emotional social support

The 10-item emotional support and 12-item guidance subscales of the Social Support Behaviors Inventory (SS-B) assessed parent/friend support (1 = not at all, 5 = about every day, Doty et al., 2010, Vaux et al., 1987). Example item: “How often have my parents/friends…comforted me when I was upset.”

#### 2.3.4 General supportive communication

The Inventory of Parents-Peer Attachment (IPPA) 8-item subscale for communication assessed quality of general communication between the participant and parents/friends (1 = almost never or never true, 5 = almost always or always true; Armsden and Greenberg, 1987). Example item: “My parents/friends sense when I’m upset about something.”

#### 2.3.5 Sex communication

The Parent–Child Sex Communication Inventory (PCSCI) assessed aspects of sex communication quality between participants and parents/friends, including perceptions of how open, comfortable, and respectful the communication is (Openness of Sex Communication subscale [5 items; 1 = strongly disagree, 5 = strongly agree]), as well as the degree to which participants feel they need to withhold information about their sexual behavior during communication (Lack of Sex Communication subscale [3 items; 1 = never, 5 = once per week or more]); Rogers et al., 2022; Busby et al., 2023). Examples items: “I find it easy to discuss sex with my parents/friends” and “How often do you keep secrets from my parents/friends about my sexual behaviors with others.”

#### 2.3.6 Drinking in sexual contexts (DISC)

Questions assessed drinking during dating, sexual contact, and intercourse, measuring frequency and quantity of alcohol consumed in each of these sexual contexts (Jacques-Tiura et al., 2007; Zawacki et al., 2003). Frequency was assessed using a 6-point scale (1 = never, 6 = nearly every time or every time) and amount was assessed on an 8-point scale (1 = none, 8 = thirteen or more drinks). Frequency and quantity items were multiplied for each context (dating, contact, intercourse) and averaged to create the DISC variable.

Study measures were selected based on psychometrics and validity in prior sexual health research (SS-B, Doty et al., 2010; IPPA, Therriault et al., 2024; PCSCI, Busby et al., 2023; DISC, Jacques-Tiura et al., 2007).

#### 2.3.7 Potential covariates

Potential covariates were assessed that influenced drinking behavior, sexual health risk, and social support in past research but were not the focus of the present study so their confounding with SGM identity could be controlled in path analyses. One item assessed participants’ socio-economic standing, asking where their family would rank when they were growing up (1–10, 10 being those “who are the best off, those who have the most money, most education, and best jobs”; Adler et al., 2000). One item assessed parent religious influence, asking participants to “indicate how influential your parents’ religious beliefs and backgrounds were in your life” (1 = not at all, 7 = extremely).

### 2.4 Data analysis

Path analyses were conducted using the PROCESS macro (Hayes, 2022) in SPSS. Two mediation path models (Model 4, 5,000 bootstrap resamples) were specified, one examining parent variables, and the other examining friend variables. In order to maintain adequate power, we chose to analyze friend and parent variables in two separate models rather than one combined model requiring an N of 791 according to power analysis. This analysis choice, however, limited our ability to adjust for and directly compare cross-source correlations within each model. The two models contained the same predictor, outcome, and covariates. Each path model specified one predictor variable (X = CH or SGM), four parallel mediator variables (M1 = support; M2 = general communication; M3 = openness of sex communication; M4 = lack of sex communication), and one outcome variable (Y = DISC). The covariates of ethnicity, socio-economic standing, and parent religious influence were included in tests of all direct (a, b) and indirect (ab) paths in both path models.

## 3 Results

### 3.1 Preliminary analyses

Power analysis indicated that the minimum sample size needed for a path analysis with one predictor and four mediators in each model (*p* < 0.05, *beta* = 0.80, *d* = 0.02) (Li et al., 2022; Henrich et al., 2006) was 647 (Faul et al., 2009).

Data were checked and cleaned in compliance with assumptions for path analysis using OLS regression. Scores on multi-item measures were computed using available-case imputation so that each participant’s score was comprised of their own answered items to address missing data. Covariate assessment indicated that compared to CH participants, SGM participants reported significantly lower socio-economic standing, (SGM: *M* = 5.28, SD = 1.88, CH: *M* = 5.79, SD = 1.92), *F*(1,672) = 8.91, *p* = 0.003; lower parent religious influence, (SGM: *M* = 4.12, SD = 1.58, CH: *M* = 4.64, SD = 1.75), *F*(1,672) = 11.78, *p* < 0.001; and were more likely to report the ethnic identities of White *X*^2^(1,674) = 6.14, *p* = 0.013 and multi-ethnic, *X*^2^(1,674) = 4.48, *p* = 0.048.

### 3.2 Path model 1-parent support and communication (*N* = 674)

A path diagram including all parent variables is depicted in Figure 1. The total path coefficient from SGM identity to DISC was not significant, *b* = 0.14, SE = 0.26, 95% CI [−0.38, 0.65], *p* = 0.605. There were significant, negative paths between SGM identity and the variables of parent support, *b* = −0.15, SE = 0.08, 95% CI [−0.30, −0.01], *p* = 0.041, and parent general communication, *b* = −0.30, SE = 0.09, 95% CI [−0.48, −0.13], *p* < 0.001. However, neither parent support, *b* = −0.07, SE = 0.21, 95% CI [−0.47, 0.34], *p* = 0.747, nor parent general communication, *b* = 0.09, SE = 0.18, 95% CI [−0.28, 0.45], *p* = 0.644, were significantly associated with DISC. There were no significant associations of parent open sex communication with SGM, *b* = −0.07, SE = 0.10, 95% CI [−0.26, 0.12], *p* = 0.484, or with DISC, *b* = −0.08, SE = 0.12, 95% CI [−0.32, 0.16], *p* = 0.506. Indirect paths were not significant via parent support, *b* = 0.01, SE = 0.04, 95% CI [−0.05, 0.11], parent general communication, *b* = −0.03, SE = 0.06, 95% CI [−0.16, 0.08], and parent open sex communication, *b* = 0.01, SE = 0.02, 95% CI [−0.01, 0.06]. The variable of parent lack of sex communication showed a significant, positive association with SGM identity, b = 0.68, SE = 0.14, 95% CI [0.40, 0.96], *p* < 0.001, and with DISC, *b* = 0.15, SE = 0.07, 95% CI [0.01, 0.30], *p* = 0.039. The indirect path from SGM to DISC through parent lack of sex communication was significant, *b* = 0.11, SE = 0.06, 95% CI [0.01, 0.24].

**Figure 1:**
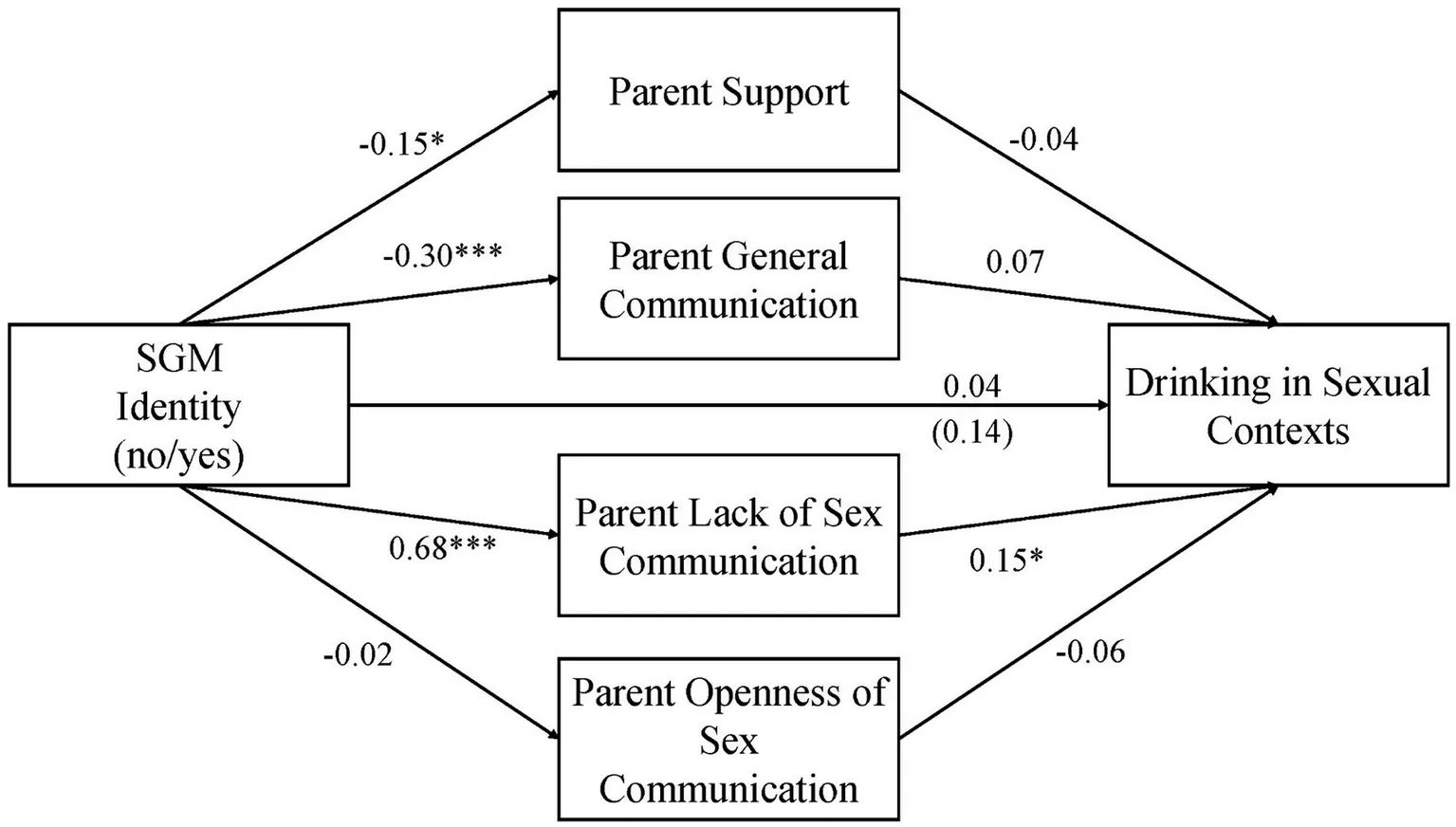
Path analysis model of the associations among sexual and gender identity, parent support variables, and drinking in sexual contexts. Path values presented are the unstandardized linear regression coefficients. All paths were controlled for ethnicity, socio-economic standing, and parent religious influence. ^*^p < 0.05, ^**^p < 0.01, ^***^p < 0.001, () indicates the total path coefficient of SGM Identity to Drinking in Sexual Contexts. SGM, sexual and gender minority.

### 3.3 Path model 2-friend support and communication (N = 674)

A path diagram including all friend variables is presented in Figure 2. The total path coefficient from SGM identity to DISC was not significant, *b* = 0.14, SE = 0.26, 95% CI [−0.38, 0.65], *p* = 0.605. Friend support was not significantly associated with either SGM identity, *b* = 0.07, SE = 0.07, 95% CI [−0.07, 0.21], *p* = 0.337, or with DISC, *b* = 0.05, SE = 0.23, 95% CI [−0.41, 0.50], *p* = 0.845. Friend general communication showed no significant association with either SGM identity, *b* = 0.12, SE = 0.07, 95% CI [−0.03, 0.26], *p* = 0.118, or DISC, *b* = −0.19, SE = 0.22, 95% CI [−0.63, 0.25], *p* = 0.395. Friend lack of sex communication was not significantly associated with either SGM identity, *b* = 0.18, SE = 0.11, 95% CI [−0.04, 0.40], *p* = 0.116, or with DISC, *b* = 0.09, SE = 0.09, 95% CI [−0.09, 0.26], *p* = 0.335. Indirect paths were not significant via friend support, *b* = 0.00, SE = 0.02, 95% CI [−0.03, 0.06], friend general communication, *b* = −0.02, SE = 0.03, 95% CI [−0.13, 0.02], and friend lack of sex communication, *b* = 0.02, SE = 0.02, 95% CI [−0.01, 0.09]. Friend open sex communication showed a significant, positive association with SGM identity, *b* = 0.27, SE = 0.08, 95% CI [0.11, 0.43], *p* < 0.001, and with DISC, *b* = 0.40, SE = 0.14, 95% CI [0.13, 0.67], *p* = 0.004. The indirect path from SGM identity to DISC through friend open sex communication was significant, *b* = 0.11, SE = 0.04, 95% CI [0.04, 0.22].

**Figure 2:**
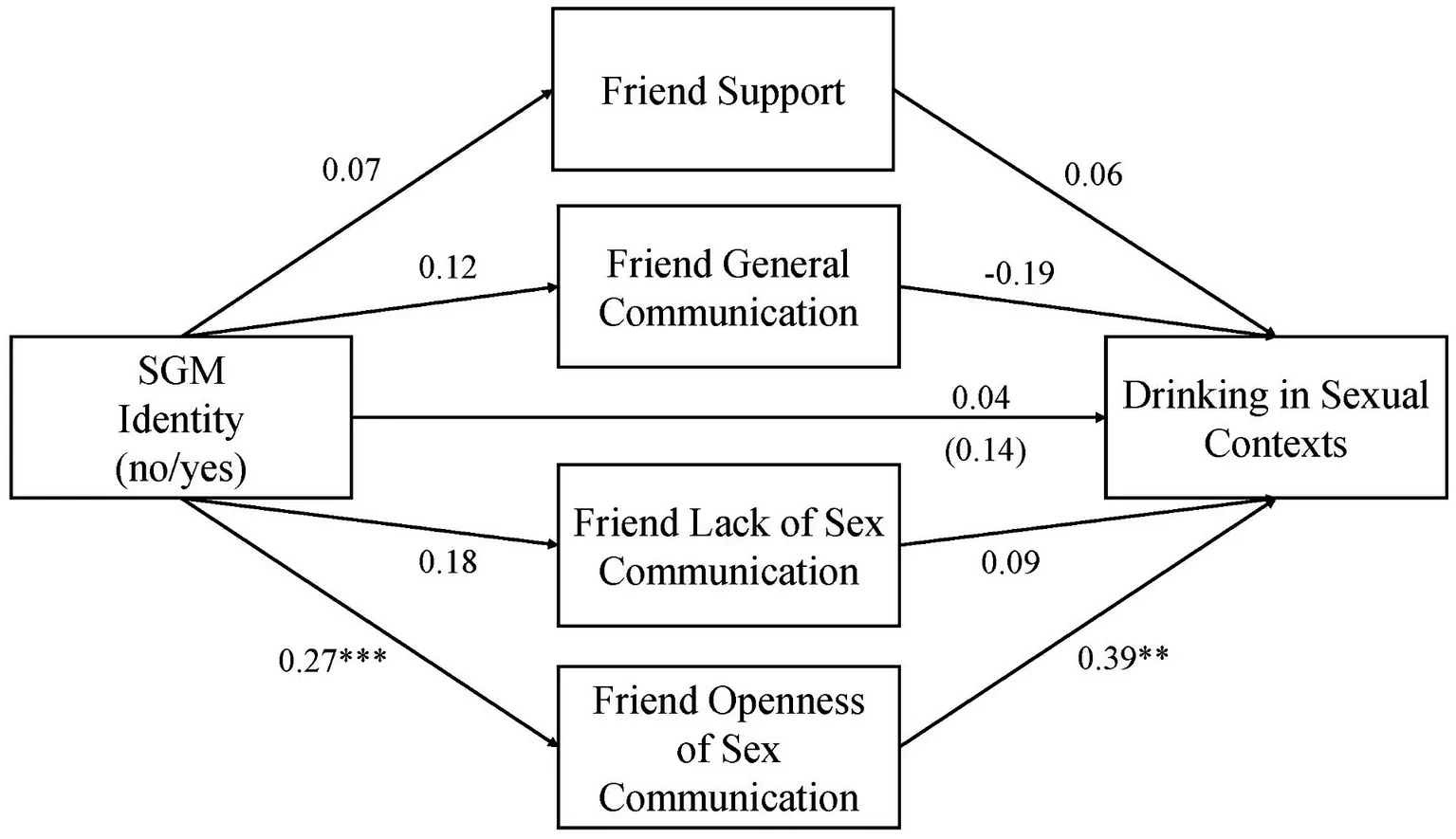
Path analysis model of the associations among sexual and gender identity, friend support variables, and drinking in sexual contexts. Path values presented are the unstandardized linear regression coefficients. All paths were controlled for ethnicity, socio-economic standing, and parent religious influence. ^*^p < 0.05, ^**^p < 0.01, ^***^p < 0.001, () indicates the total path coefficient of SGM Identity to Drinking in Sexual Contexts. SGM, sexual and gender minority.

## 4 Discussion

### 4.1 Key findings

This study found that participants’ SGM identity was related to greater alcohol use in sexual situations through its association with aspects of sex communication, but not general communication and support, provided by parents and friends. While the total path from SGM identity to DISC may have been diluted by the presence of both positive and negative indirect paths within the model (Hayes, 2022), parent lack of sex communication and friend open sex communication emerged as significant individual variables in the relationship between SGM and DISC. As hypothesized, compared to CH participants, SGM participants were more likely to report a lack of sex communication with parents and, in turn, lack of parental sex communication was associated with significantly higher levels of DISC. While SGM participants reported lower quantity (a lack) of sex communication than CH participants, they did not report lower quality in terms of openness. This finding supports and extends existing literature by identifying lack of sex communication with parents as a possible specific point of communication disruption.

In contrast to parent sex communication, differences emerged for quality but not quantity of friend sex communication. Friend openness of sex communication was significantly higher among SGM individuals than CH participants and, unexpectedly, was associated with greater DISC. It is possible that the content of friends’ open sex communication may have communicated permissive sexual and drinking attitudes that correlate with higher alcohol use in sexual contexts (Jessor, 1991; Watts et al., 2023). Future research should examine how the content of friend attitudes influences the relationship between sex communication and DISC. Research should also assess the sexual and gender identity of friends who provide support, as some research suggests beneficial relationships of identity-specific support from SGM peers with substance use and sexual risk (Jamil et al., 2009; Ott et al., 2022; Caba et al., 2024).

Consistent with past research, SGM participants reported experiencing significantly lower levels of general support and communication from parents than did CH participants (Robinson, 2018; Ryan et al., 2010). Lower parental support and communication, however, were not significantly associated with DISC. Different providers of support were also examined, building on past research suggesting that friend support and communication play a more important role in SGM adolescent’s sexual expression than does parent support (Doty et al., 2010; Jamil et al., 2009). However, results indicated no significant differences in SGM and CH friend support and general communication, nor were friend support and general communication significantly related to DISC.

Parent variable results suggest that parent lack of sex communication may be particularly relevant to SGM individual’s alcohol-involved sexual behavior. Within a Minority Stress Theory framework, these results suggest that lack of parent sex communication may represent a distinct form of minority stress for SGM individuals. From a social support perspective, “optimal matching effects,” where lacking the support most closely addressing a health risk behavior is associated with the largest increases in it, may have contributed to the significant association between lack of parent sex communication and increased drinking in sexual situations (Cutrona, 1990; Moon et al., 2019).

### 4.2 Limitations and future directions

This cross-sectional study cannot determine temporal order of variables. While our results may reflect that DISC behaviors follow parent lack sex communication, it is possible that DISC may precede a lack of sex communication with parents. Analyses included numerous tests without correction for risk of Type 1 error and the confidence intervals for both significant indirect paths neared zero, which increases the possibility of a false positive. The parent and friend path models were run separately to enhance power. Despite this analytic choice, we were able to address our study aims to examine the understudied topic of alcohol use during sexual encounters among SGM emerging adults, investigate the roles of receiving different types of support and communication, and specify friends and parents as sources of support. Because of this analytic choice, however, we were unable to address our study aim of investigating whether friend support compensates for parent support among SGM participants because the correlations among parent and friend support could not be considered.

Our sample consisted of college students largely under the legal drinking age from a single university, limiting the generalizability of results beyond these groups. Size differences in SGM identity categories in the sample may mean that the data is more reflective of the experiences of some SGM identity groups than others. Despite these limitations, the novel results in this preliminary study may provide groundwork for larger studies that can focus on specific groups within the SGM population, incorporate a broader range of potential mechanisms, and examine temporal precedence of variables using prospective methods. Other avenues include incorporating relevant processes such as concealment of SGM identity, and asking participants to report precise number of drinks consumed during sexual events to provide clinically relevant information regarding how much additional drinking significant associations represent.

## 5 Conclusion

This study’s novel results shed light on the potential roles of parent and friend sex communication in the relationship between SGM identity and DISC, an important yet understudied factor in adverse health outcomes among SGM emerging adults. These results also suggest specific types of sex communication with SGM emerging adults that appear to function differently in their relationship with sexual risk-related behavior depending on whether the communication source is parents or friends. Our findings emphasize the importance of DISC within the conversation of SGM sexual health and underscore the need for family and peer-based interventions.

## Data Availability

The raw data supporting the conclusions of this article will be made available by the authors, without undue reservation.

## References

[ref1] Abern L. Diego D. Krempasky C. Cook J. Maguire K. (2022). Prevalence of sexual assault in a cohort of transgender and gender diverse individuals. J. Gen. Intern. Med. 38, 1331–1333. doi: 10.1007/s11606-022-07900-y, 36357726 PMC10110792

[ref2] Adler N. E. Epel E. S. Castellazzo G. Ickovics J. R. (2000). Relationship of subjective and objective social status with psychological and physiological functioning: preliminary data in healthy, white women. Health Psychol. 19, 586–592. doi: 10.1037//0278-6133.19.6.586, 11129362

[ref3] Armsden G. C. Greenberg M. T. (1987). The inventory of parent and peer attachment: individual differences and their relationship to psychological well-being in adolescence. J. Youth Adolesc. 16, 427–454. doi: 10.1007/BF02202939, 24277469

[ref4] Berry M. S. Johnson M. W. (2018). Does being drunk or high cause HIV sexual risk behavior? A systematic review of drug administration studies. Pharmacol. Biochem. Behav. 164, 125–138. doi: 10.1016/j.pbb.2017.08.009, 28843425 PMC5747990

[ref5] Bond K. T. Mansour M. Kershaw T. Flores D. D. (2023). “Whenever you’re ready to talk about it”: prompts and barriers to inclusive family sex communication with gay, bisexual, and queer sons. Am. J. Sex. Educ. 18, 569–593. doi: 10.1080/15546128.2022.2141935, 38213905 PMC10783024

[ref6] Bouris A. Hill B. J. Fisher K. Erickson G. Schneider J. A. (2015). Mother–son communication about sex and routine human immunodeficiency virus testing among younger men of color who have sex with men. J. Adolesc. Health 57, 515–522. doi: 10.1016/j.jadohealth.2015.07.007, 26321527 PMC4624009

[ref7] Bruederle A. Delany-Moretlwe S. Mmari K. Brahmbhatt H. (2019). Social support and its effects on adolescent sexual risk taking: a look at vulnerable populations in Baltimore and Johannesburg. J. Adolesc. Health 64, 56–62. doi: 10.1016/j.jadohealth.2018.07.004 30287132

[ref8] Busby D. M. Jeffery N. R. Hanna-Walker V. R. (2023). Parental sexual passion and parent-child communication about sex. J. Child Fam. Stud. 32, 2419–2430. doi: 10.1007/s10826-022-02369-z

[ref9] Caba A. E. Fish J. N. Wheldon C. W. Watson R. J. (2024). The association of stigma, school, and family factors with patterns of substance use among LGBTQ youth. Prev. Sci. 25, 948–962. doi: 10.1007/s11121-024-01703-9, 38970724

[ref10] Canan S. N. Jozkowski K. N. Wiersma-Mosley J. D. Bradley M. Blunt-Vinti H. (2021). Differences in lesbian, bisexual, and heterosexual women’s experiences of sexual assault and rape in a national U.S. sample. J. Interpers. Violence 36, 9100–9120. doi: 10.1177/0886260519863725, 31347442

[ref11] Centers for Disease Control and Prevention. (2024a). Fast facts: HIV and gay and bisexual men. Available online at: www.cdc.gov/hiv/data-research/facts-stats/gay-bisexual-men.html (accessed May 19, 2026).

[ref12] Centers for Disease Control and Prevention (2024b). Fast facts: HIV and transgender people. Available online at: www.cdc.gov/hiv/data-research/facts-stats/transgender-people.html (accessed May 19, 2026).

[ref13] Centers for Disease Control and Prevention (2024c). Youth risk behavior survey: Data summary & trends report 2013–2023 Atlanta, GA. Available online at: https://www.cdc.gov/yrbs/index.html (accessed May 19, 2026).

[ref14] Collins R. L. Parks G. A. Marlatt G. A. (1985). Social determinants of alcohol consumption: the effects of social interaction and model status on the self-administration of alcohol. J. Consult. Clin. Psychol. 53, 189–200. doi: 10.1037/0022-006X.53.2.189, 3998247

[ref15] Coulter R. W. Mair C. Miller E. Blosnich J. R. Matthews D. D. McCauley H. L. (2017). Prevalence of past-year sexual assault victimization among undergraduate students: exploring differences by and intersections of gender identity, sexual identity, and race/ethnicity. Prev. Sci. 18, 726–736. doi: 10.1007/s11121-017-0762-8, 28210919 PMC5511765

[ref16] Cutrona C. E. (1990). Stress and social support - in search of optimal matching. J. Soc. Clin. Psychol. 9, 3–14. doi: 10.1521/jscp.1990.9.1.3

[ref17] Doty N. D. Willoughby B. L. Lindahl K. M. Malik N. M. (2010). Sexuality related social support among lesbian, gay, and bisexual youth. J. Youth Adolesc. 39, 1134–1147. doi: 10.1007/s10964-010-9566-x, 20593304

[ref18] Dutra R. Miller K. S. Forehand R. (1999). The process and content of sexual communication with adolescents in two-parent families: associations with sexual risk-taking behavior. AIDS Behav. 3, 59–66. doi: 10.1023/a:1025419519668

[ref19] Eisenberg M. E. Lust K. Mathiason M. A. Porta C. M. (2021). Sexual assault, sexual orientation, and reporting among college students. J. Interpers. Violence 36, 62–82. doi: 10.1177/0886260517726414 29294876

[ref20] Faul F. Erdfelder E. Buchner A. Lang A. G. (2009). Statistical power analyses using G*power 3.1: tests for correlation and regression analyses. Behav. Res. Methods 41, 1149–1160. doi: 10.3758/BRM.41.4.1149 19897823

[ref21] Feinstein B. A. Thomann M. Coventry R. Macapagal K. Mustanski B. Newcomb M. E. (2017). Gay and bisexual adolescent boys’ perspectives on parent–adolescent relationships and parenting practices related to teen sex and dating. Arch. Sex. Behav. 47, 1825–1837. doi: 10.1007/s10508-017-1057-7, 29280027 PMC6019640

[ref22] Flores D. D. Meanley S. P. Wood S. M. Bauermeister J. A. (2020). Family characteristics in sex communication and social support: implications for emerging adult men who have sex with men’s prep engagement. Arch. Sex. Behav. 49, 2145–2153. doi: 10.1007/s10508-020-01648-4, 32222853 PMC7781437

[ref23] Ford C. A. Pool A. C. Kahn N. F. Jaccard J. Halpern C. T. (2023). Associations between mother-adolescent and father-adolescent relationships and young adult health. JAMA Netw. Open 6:e233944. doi: 10.1001/jamanetworkopen.2023.3944, 36943264 PMC10031392

[ref24] Ghosh A. (2020). After coming out: parental acceptance of young lesbian and gay people. Sociol. Compass 14:e12740. doi: 10.1111/soc4.12740

[ref25] Glick S. N. Golden M. R. (2014). Early male partnership patterns, social support, and sexual risk behavior among young men who have sex with men. AIDS Behav. 18, 1466–1475. doi: 10.1007/s10461-013-0678-7, 24356869 PMC4065217

[ref26] Guilamo-Ramos V. Benzekri A. Thimm-Kaiser M. (2023). Evaluating the efficacy of an online, family-based intervention to promote adolescent sexual health: a study protocol for a randomized controlled trial. Trials 24:181. doi: 10.1186/s13063-023-07205-3, 36906589 PMC10008101

[ref27] Hayes A. F. (2022). Introduction to Mediation, Moderation, and Conditional process Analysis: A Regression-based Approach. 3rd Edn New York City, NY: The Guildford Press.

[ref28] Henrich C. C. Brookmeyer K. A. Shrier L. A. Shahar G. (2006). Supportive relationships and sexual risk behavior in adolescence: an ecological–transactional approach. J. Pediatr. Psychol. 31, 286–297. doi: 10.1093/jpepsy/jsj024, 15827352

[ref29] Hinds Z. Herbitter C. Bryant W. T. Newberger N. G. Livingston N. A. (2024). Hazardous substance use among sexual and gender minority adults: a deeper look into distal minority stressors. Psychol. Sex. Orientat. Gend. Divers. 11, 105–112. doi: 10.1037/sgd0000583, 37885566 PMC10601395

[ref30] Ison J. Hindes S. Fileborn B. (2025). LGBTQ+ adult sexual violence critical scoping review: victimization risk factors. Trauma Violence Abuse 27, 319–330. doi: 10.1177/15248380241311930, 39840706 PMC12953674

[ref31] Jacques-Tiura A. J. Abbey A. Parkhill M. R. Zawacki T. (2007). Why do some men misperceive women’s sexual intentions more frequently than others do? An application of the confluence model. Personal. Soc. Psychol. Bull. 33, 1467–1480. doi: 10.1177/0146167207306281, 17933743 PMC4484569

[ref32] James S. Herman J. Rankin S. Keisling M. Mottet L. Anafi M. A. (2016). The report of the 2015 US transgender survey. Washington, DC: National Center for Transgender Equality. Available online at: http://hdl.handle.net/20.500.11990/1299 (Accessed September 21, 2026).

[ref33] Jamil O. B. Harper G. W. Fernandez M. I. (2009). Sexual and ethnic identity development among gay–bisexual–questioning (GBQ) male ethnic minority adolescents Cultur. Divers. Ethnic Minor. Psychol. 15, 203–214. doi: 10.1037/a0014795 PMC2846409 19594249

[ref34] Jessor R. (1991). Risk behavior in adolescence: a psychosocial framework for understanding and action. J. Adolesc. Health 12, 597–605. doi: 10.1016/1054-139X(91)90007-K, 1799569

[ref35] Kammer-Kerwick M. Wang A. McClain T. Hoefer S. Swartout K. M. Backes B. . (2021). Sexual violence among gender and sexual minority college students: the risk and extent of victimization and related health and educational outcomes. J. Interpers. Violence 36, 10499–10526. doi: 10.1177/0886260519883866, 31686584

[ref36] Kiekens W. J. Baams L. Fish J. N. Watson R. J. (2021). Associations of relationship experiences, dating violence, sexual harassment, and assault with alcohol use among sexual and gender minority adolescents. J. Interpers. Violence 37, NP15176–NP15204. doi: 10.1177/08862605211001469, 33719695 PMC8917816

[ref37] Li J. J. Hyun S. Stevens C. Chen J. A. Liu C. H. (2022). Binge drinking and sexual risk behavior among US sexual and gender minority college students. Am. J. Addict. 31, 22–30. doi: 10.1111/ajad.13244, 34907619

[ref38] Marcantonio T. L. Willis M. (2022). Examining substance-involved sexual experiences and consent communication by sexual identity. Psychol. Theol. Sex. 14, 279–293. doi: 10.1080/19419899.2022.2106884, 37339054

[ref39] McKay E. A. Fontenot H. B. (2020). Parent-adolescent sex communication with sexual and gender minority youth: an integrated review. J. Pediatr. Health Care 34, e37–e48. doi: 10.1016/j.pedhc.2020.04.004, 32861429

[ref40] Moon T. Mathias C. W. Mullen J. Karns-Wright T. E. Hill-Kapturczak N. Roache J. D. . (2019). The role of social support in motivating reductions in alcohol use: a test of three models of social support in alcohol-impaired drivers. Alcohol. Clin. Exp. Res. 43, 123–134. doi: 10.1111/acer.13911, 30431660 PMC6380519

[ref41] Ndlovu R. Moyo P. L. (2026). Exploring prevalence of risky sexual behaviour among adolescents in high schools: a systematic review. SAGE Open Med. 14, 1–9. doi: 10.1177/20503121251413045, 41693751 PMC12905071

[ref42] Newcomb M. E. LaSala M. C. Bouris A. Mustanski B. Prado G. Schrager S. M. . (2019). The influence of families on LGBTQ youth health: a call to action for innovation in research and intervention development. LGBT Health. 6, 139–145. doi: 10.1089/lgbt.2018.0157, 30844341 PMC6551980

[ref43] Ott M. Q. Clark M. A. Balestrieri S. G. Gamarel K. E. Barnett N. P. (2022). Social networks and sexual and gender minority disparities in alcohol use and consequences among first-year college students. LGBT Health. 9, 489–495. doi: 10.1089/lgbt.2019.0225, 35727117 PMC9587779

[ref44] Read J. P. (2025). Alcohol and sexual and intimate partner violence: a review, current trends, and a look toward the future. J. Stud. Alcohol Drugs 86, 161–165. doi: 10.15288/jsad.25-00079, 40035817 PMC11980409

[ref45] Robinson B. A. (2018). Conditional families and lesbian, gay, bisexual, transgender, and queer youth homelessness: gender, sexuality, family instability, and rejection. J. Marriage Fam. 80, 383–396. doi: 10.1111/jomf.12466

[ref46] Rogers A. A. Padilla-Walker L. M. Hurst J. L. (2022). Development and testing of the parent-child sex communication inventory: a multidimensional assessment tool for parent and adolescent informants. J. Sex Res. 59, 98–111. doi: 10.1080/00224499.2020.1792398, 32660280

[ref47] Ryan C. Russell S. T. Huebner D. Diaz R. Sanchez J. (2010). Family acceptance in adolescence and the health of LGBT young adults. J. Child Adolesc. Psychiatr. Nurs. 23, 205–213. doi: 10.1111/j.1744-6171.2010.00246.x, 21073595

[ref48] Saint-Eloi C. H. Hutchinson M. K. Sutherland M. A. (2024). The influence of pre-college behaviors and parenting practices on alcohol misuse, sexual risk-taking, and adverse outcomes among first-year college women. J. Am. Coll. Heal. 72, 731–742. doi: 10.1080/07448481.2022.2056416, 35472005

[ref49] Scroggs B. Rosenberger J. G. (2024). Understanding the relationship between casual substance use and sexual risk behaviors in sexual and gender minority emerging adults. Sexual. Gend. Divers. Soc. Serv. 36, 160–169. doi: 10.1080/10538720.2023.2246924, 37339054

[ref50] Shuper P. A. Joharchi N. Monti P. M. Loutfy M. Rehm J. (2017). Acute alcohol consumption directly increases HIV transmission risk: a randomized controlled experiment. J. Acquir. Immune Defic. Syndr. 76, 493–500. doi: 10.1097/QAI.0000000000001549, 28930769 PMC5680134

[ref51] Steiner R. J. Sheremenko G. Lesesne C. Dittus P. J. Sieving R. E. Ethier K. A. (2019). Adolescent connectedness and adult health outcomes. Pediatrics 144:e20183766. doi: 10.1542/peds.2018-3766, 31235609 PMC9125410

[ref52] Therriault D. Lemelin J. Toupin J. Déry M. (2024). Peer attachment in adolescence: what are the individual and relational associated factors? Soc. Dev. 33:e12762. doi: 10.1111/sode.12762

[ref53] Thoma B. C. Huebner D. M. (2014). Parental monitoring, parent–adolescent communication about sex, and sexual risk among young men who have sex with men. AIDS Behav. 18, 1604–1614. doi: 10.1007/s10461-014-0717-z, 24549462 PMC4108561

[ref54] Vaux A. Riedel S. Stewart D. (1987). Modes of social support: the social support behaviors (SS-B) scale. Am. J. Community Psychol. 15, 209–232. doi: 10.1007/BF00919279

[ref55] Watts L. L. Hamza E. A. Bedewy D. A. Moustafa A. A. (2023). A meta-analysis study on peer influence and adolescent substance use. Curr. Psychol. 43, 3866–3881. doi: 10.1007/s12144-023-04944-z

[ref56] Widman L. Evans R. Javidi H. Choukas-Bradley S. (2019). Assessment of parent-based interventions for adolescent sexual health: a systematic review and meta-analysis. JAMA Pediatr. 173, 866–877. doi: 10.1001/jamapediatrics.2019.2324, 31355860 PMC6664375

[ref57] Xu W. Wheeler L. A. Littleton H. Kim S. Thomas R. Gardella J. . (2026). A prospective study examining the consequences of family rejection for sexual and gender minority youth. Fam. Relat. 1–16. doi: 10.1111/fare.70286

[ref58] Yeo G. Lansford J. E. Rudolph K. D. (2025). How does perceived social support relate to human thriving? A systematic review with meta-analyses. Psychol. Bull. 151, 1089–1124. doi: 10.1037/bul0000491, 41100292

[ref59] Zawacki T. Abbey A. Buck P. O. McAuslan P. Clinton-Sherrod A. M. (2003). Perpetrators of alcohol-involved sexual assaults: how do they differ from other sexual assault perpetrators and nonperpetrators? Aggress. Behav. 29, 366–380. doi: 10.1002/ab.10076, 26430287 PMC4586126

